# Evaluating the Oxidation Rate of Reduced Ferredoxin in *Arabidopsis thaliana* Independent of Photosynthetic Linear Electron Flow: Plausible Activity of Ferredoxin-Dependent Cyclic Electron Flow around Photosystem I

**DOI:** 10.3390/ijms241512145

**Published:** 2023-07-29

**Authors:** Miho Ohnishi, Shu Maekawa, Shinya Wada, Kentaro Ifuku, Chikahiro Miyake

**Affiliations:** 1 Graduate School for Agricultural Science, Kobe University, 1-1 Rokkodai, Nada-Ku, Kobe 657-8501, Japan; 2 Core Research for Evolutional Science and Technology (CREST), Japan Science and Technology Agency (JST), 7 Gobancho, Tokyo 102-0076, Japan; 3 Graduate School for Agriculture, Kyoto University, Kitashirakawa Oiwake-cho, Sakyo-ku, Kyoto 606-8502, Japan

**Keywords:** cyclic electron flow, ferredoxin, NADH dehydrogenase, pgr5, photosynthesis, photosystem I

## Abstract

The activity of ferredoxin (Fd)-dependent cyclic electron flow (Fd-CEF) around photosystem I (PSI) was determined in intact leaves of *Arabidopsis thaliana*. The oxidation rate of Fd reduced by PSI (vFd) and photosynthetic linear electron flow activity are simultaneously measured under actinic light illumination. The vFd showed a curved response to the photosynthetic linear electron flow activity. In the lower range of photosynthetic linear flow activity with plastoquinone (PQ) in a highly reduced state, vFd clearly showed a linear relationship with photosynthetic linear electron flow activity. On the other hand, vFd increased sharply when photosynthetic linear electron flow activity became saturated with oxidized PQ as the net CO_2_ assimilation rate increased. That is, under higher photosynthesis conditions, we observed excess vFd resulting in electron flow over photosynthetic linear electron flow. The situation in which excess vFd was observed was consistent with the previous Fd-CEF model. Thus, excess vFd could be attributed to the in vivo activity of Fd-CEF. Furthermore, the excess vFd was also observed in NAD(P)H dehydrogenase-deficient mutants localized in the thylakoid membrane. The physiological significance of the excessive vFd was discussed.

## 1. Introduction

In photosynthesis, both reaction center chlorophylls (P680 in photosystem II (PSII) and P700 in photosystem I (PSI)) are excited by the photon energy absorbed by the light-harvesting systems located in PSII/I. The absorbed photon energy is converted to the electron flux starting at H_2_O oxidation in PSII, and the electron flow ends in the reduction of ferredoxin (Fd) at PSI. The reduced Fd delivers electrons mainly to reactions catalyzed by Fd-NADP oxidoreductase to produce NADPH. Simultaneously, with the photosynthetic linear electron flow from H_2_O to Fd, protons accumulate on the luminal side of the thylakoid membrane, forming ΔpH across the membrane. The ΔpH, as a proton motive force, drives ATP synthase to produce ATP. These energy compounds, including reduced Fd, NADPH, and ATP produced in the light reaction, drive the dark reactions, net CO_2_ assimilation, and photorespiration in C_3_ plants.

Many researchers have proposed that Fd could also deliver electrons to plastoquinone (PQ) in the photosynthetic electron transport system through Fd-quinone oxidoreductase (FQR) [1,2,3]. FQR-dependent electron flow has been called Fd-dependent cyclic electron flow around PSI (Fd-CEF). If the process works, Fd-CEF could contribute to the induction of ΔpH across the thylakoid membrane [2,4,5]. The electron flow from the reduced Fd to PQ through the cytochrome (Cyt) *b*_6_/*f* complex could drive excess Q-cycle activity in the Cyt *b*_6_/*f* complex against the photosynthetic linear electron flow and form an excessive ΔpH. The ΔpH induced by Fd-CEF could contribute to the induction of nonphotochemical quenching of chlorophyll (Chl) fluorescence and the production of ATP [2,5].

In vivo, the activity of Fd-CEF has been shown as the excess quantum yield of PSI against the apparent quantum yield of PSII [Y(II)] [6,7]. The apparent quantum yield of PSI [Y(I)] becomes excessive against Y(II) with oxidized P700 [8]; these observations were artifacts [9]. The value of Y(I) was measured by the saturation-pulse illumination method [10]. The saturation-pulse illumination under actinic light illumination excites the ground state of P700 to oxidized P700 (P700^+^) through light-excited P700 (P700*), and the ratio of induced P700^+^ to total P700 has been estimated as Y(I). The amount of induced P700^+^ depends on the rate-determining step of the P700 photooxidation reduction cycle in PSI [9]. In the P700 photooxidation reduction cycle, P700* is oxidized to P700^+^ by donating electrons to the electron acceptors A_o_, A_1_, F_x_, and F_A_/F_B_ sequentially to Fd. P700^+^ is reduced to the ground state of P700 by electrons from PSII through PQ, the Cyt *b*_6_/*f* complex, and plastocyanin (PC). If the reduction of P700^+^ in the cycle was the rate-determining step, in which oxidized P700 accumulated under actinic light illumination, the amount of the ground state of P700 was overestimated by the saturation-pulse illumination method [9]. On the other hand, if the oxidation of P700* in the cycle was the rate-determining step, the amount of the ground state of P700 was underestimated by the saturation-pulse illumination method [10]. It is too difficult to estimate the true Y(I) when evaluating Fd-CEF activity [9].

Furthermore, the ability of electron donation from the reduced Fd to the oxidized PQ through FQR has been evaluated using the isolated thylakoid membrane [3]. The addition of Fd/NADPH to the thylakoid membrane increased the minimal yield of Chl fluorescence, and PQ was reduced by the reduced Fd. The rate of increase in the minimal yield of Chl fluorescence was treated as the activity of FQR. However, the reduced Fd donates electrons to PSII, not PQ [11]. Furthermore, the reduced Fd donates electrons to Cyt *b*_559_, which is inhibited by antimycin A [12]. The effect of antimycin A also inhibits Fd-dependent quenching of 9-aminoacridine fluorescence, which is driven by far-red light illumination [12]. The quenching of 9-aminoacridine fluorescence shows ΔpH formation across the thylakoid membrane. Far-red-driven quenching also results from the activity of Fd-CEF. However, as described above, electron donation from the reduced Fd to PSII could also induce the quenching of 9-aminoacridine fluorescence by maintaining the photosynthetic linear electron flow.

As described above, no credible methods are available to detect and evaluate Fd-CEF activity, that is, FQR activity. To elucidate the physiological function of Fd-CEF, an assay system capable of detecting Fd-CEF in vivo was required. In the present research, we monitored the redox reaction of Fd simultaneously with Chl fluorescence, P700^+^ and PC^+^ absorbance changes, and net CO_2_ assimilation using intact leaves of *Arabidopsis thaliana*. As a result, excessive turnover of the Fd redox reaction was observed, which is not explained by the photosynthetic linear electron flow in vivo. The excessive Fd redox reaction, which followed the model of Fd-CEF activity, was characterized [1]. The redox balance between the electron donor (the reduced Fd) and the electron acceptor (the oxidized PQ) is needed to obtain the maximum Fd-CEF activity, which is a possible reason why we did not detect the excessive Fd redox reaction [13]. During limited photosynthesis, in which PQ was highly reduced and the apparent quantum yield of PSII was low, the excessive Fd redox reaction was suppressed in vivo. We proposed that the excessive Fd redox reaction reflected the activity of Fd-CEF. Next, we compared Fd-CEF activity between the wild type (WT) and an *Arabidopsis thaliana* mutant lacking NAD(P)H dehydrogenase (NDH) (*crr4*). NDH has been considered to catalyze electron donation from reduced Fd to PQ in the photosynthetic electron transport system [6,14]. *crr4* did not show an Fd-dependent increase in the minimal Chl fluorescence [15]. Surprisingly, *crr4* showed almost the same Fd-CEF activity as the wild type in vivo. The physiological function of Fd-CEF from our results was discussed.

## 2. Results

**Figure 1:**
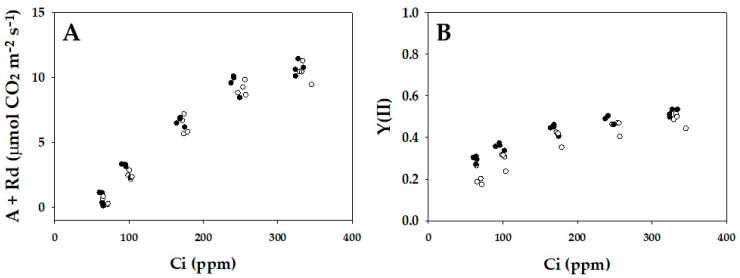
Effects of the intercellular partial pressure of CO_2_ (Ci) on the gross CO_2_ assimilation rate (A + Rd) and apparent quantum yield of photosystem II (PSII) [Y(II)] in wild-type (WT) and *crr4 Arabidopsis*. (**A**) The net CO_2_ assimilation rates were measured at 400 µmol photons m^−2^ s^−1^ and 21 kPa O_2_, and Y(II) was simultaneously measured. The dark respiration rates (Rd) were measured before starting actinic light illumination. The gross CO_2_ assimilation rates are expressed as A + Rd and were plotted against Ci. (**B**) Y(II) was plotted against Ci. The data were obtained from four independent experiments using leaves attached to four plants of both WT and *crr4* (*N* = 4). The ambient partial pressures of CO_2_ were changed from 400 ppm to 300 ppm, then 200 ppm, then 100 ppm, and finally 50 ppm at 21 kPa O_2_ for the same leaves. Black symbols, WT; White symbols, *crr4*.

Both the gross CO_2_ assimilation rate and the apparent quantum yield of PSII [Y(II)] were plotted against the intercellular partial pressures of CO_2_ (Ci) (Figure 1). These two parameters showed the same dependencies on Ci in both WT and *crr4*.

**Figure 2:**
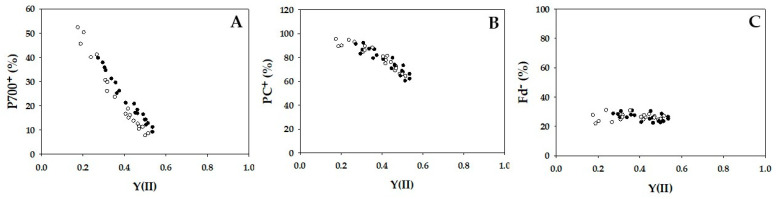
Relationships between P700^+^, PC^+^, Fd^−^, and apparent quantum yield of photosystem II (PSII) [Y(II)]. The data for each parameter were measured in the experiments depicted in Figure 1, simultaneously with the net CO_2_ assimilation rates and Y(II). (**A**) P700^+^, (**B**) PC^+^, and (**C**) Fd^−^ were plotted against Y(II). Those ratios of P700^+^, PC^+^, and Fd^−^ against the total contents are expressed. The data were obtained from four independent experiments using leaves attached to four WT and *crr4* plants (*N* = 4). Black symbols, WT; White symbols, *crr4*.

The parameters P700^+^, PC^+^, and Fd^−^ against Y(II) were plotted in Figure 2. With the decrease in Y(II) caused by lowering Ci, P700 was oxidized from approximately 10 to 40% in WT and from 10 to 50% in *crr4* (Figure 2A). Similarly, PC was oxidized from 65 to 90% in both WT and *crr4* (Figure 2B). In contrast to both P700^+^ and PC^+^, Fd^−^ did not change in response to the decrease in Y(II) in either WT or *crr4* (Figure 2C), due to the oxidation of P700 in PSI [16].

**Figure 3:**
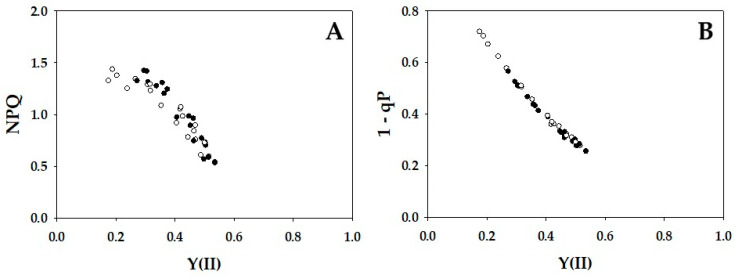
Relationships between non-photochemical quenching (NPQ), plastoquinone reduced state (1 − qP), and apparent quantum yield of photosystem II (PSII) [Y(II)]. The data for each parameter were measured in the experiments depicted in Figure 1, simultaneously with the net CO_2_ assimilation rates and Y(II). (**A**) NPQ and (**B**) 1 − qP were plotted against Y(II). The data were obtained from four independent experiments using leaves attached to four WT and *crr4* plants (*N* = 4). Black symbols, WT; White symbols, *crr4*.

The parameters non-photochemical quenching (NPQ) and plastoquinone reduced state (1 − qP) against Y(II) were plotted in Figure 3. With the decrease in Y(II), NPQ increased from approximately 0.5 to 1.5 in WT and *crr4* (Figure 3A). Increase in NPQ showed the enhancement of heat dissipation of photon energy absorbed by PSII. WT and *crr4* also showed the same dependence of 1 − qP on the decrease in Y(II), where 1 -qP increased with the decrease in Y(II) (Figure 3B). Increase in 1 − qP showed the reduction of plastoquinone pool.

**Figure 4:**
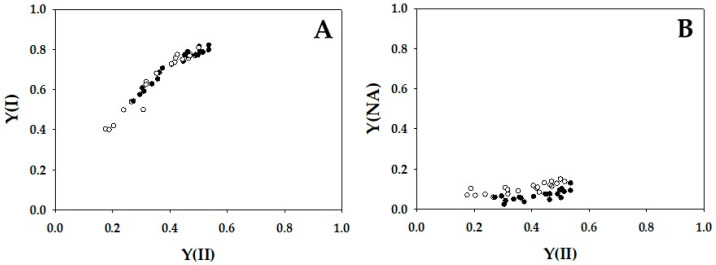
Relationships between apparent quantum yield of PSI [Y(I)], apparent quantum yield of non-photochemical energy dissipation of photoexcited P700 [Y(NA)], and apparent quantum yield of photosystem II (PSII) [Y(II)]. The data for each parameter were measured in the experiments depicted in Figure 1, simultaneously with the net CO_2_ assimilation rates and Y(II). (**A**) Y(I) and (**B**) Y(NA) were plotted against Y(II). The data were obtained from four independent experiments using leaves attached to four WT and *crr4* plants (*N* = 4). Black symbols, WT; White symbols, *crr4*.

The parameters apparent quantum yield of PSI [Y(I)] and apparent quantum yield of non-photochemically energy dissipation of photoexcited P700 [Y(NA)] were plotted in Figure 4. As described in the Introduction, Y(I) and Y(NA) were estimated by illuminating the leaves with saturated-pulse light under actinic light illumination. Y(I) reflected the strength of the donor-side limitation of the P700 photooxidation reduction cycle, and Y(NA) reflected that of the acceptor-side limitation during the saturated-pulse illumination [16]. That is, if P700 was highly oxidized under actinic light, Y(I) showed a higher value, and the reverse was also true. For example, if Y(NA) was higher, Y(I) was lower. A higher Y(NA) is accompanied by a highly reduced state of Fd [16]. With the decrease in Y(II), Y(I) decreased from approximately 0.8 to 0.5 in WT and *crr4* (Figure 4A). Y(NA) in WT was slightly lower than that in *crr4* (Figure 4B). Principally, the dependencies of Y(I) and Y(NA) on Y(II) in *crr4* were almost the same as those in WT.

**Figure 5:**
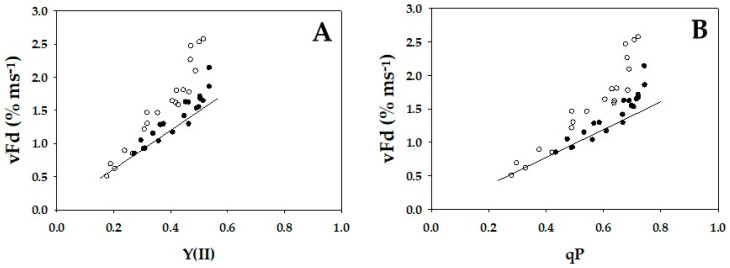
Relationships between apparent quantum yield of photosystem II (PSII) [Y(II)], plastoquinone oxidized state (qP), and vFd. The data for each parameter were measured in the experiments depicted in Figure 1, simultaneously with the net CO_2_ assimilation rates and Y(II). (**A**) Y(II) and (**B**) qP were plotted against vFd. In the experiments shown in Figure 1, the oxidation rate of ferredoxin (Fd) was determined by DIRK analysis (see Section 4). To determine the oxidation rate of Fd^−^ under illuminated conditions, actinic light was transiently turned off for 400 ms. The initial slope of the decrease in Fd^−^ indicates the oxidation rate of Fd^−^ (vFd). These data were obtained at a steady state, which was confirmed by the achievement of stable conditions for both net CO_2_ assimilation and Y(II). The data were obtained from four independent experiments using leaves attached to four WT and *crr4* plants (*N* = 4). Black symbols, WT; White symbols, *crr4*.

Finally, the oxidation rates of the reduced Fd (vFd) against Y(II) under steady-state conditions, which was estimated by DIRK analysis (see Section 4), were plotted in Figure 5. vFd did not show a linear relationship with Y(II) in either WT or *crr4* (Figure 5A). In WT, increasing Y(II) increased vFd, and a nearly linear relationship between vFd and Y(II) was found in the low range of Y(II) from 0.25 to 0.35. However, above Y(II) = 0.35, although Y(II) became saturated, vFd further increased. That is, excessive turnover of the redox reaction of Fd against Y(II) appeared in the higher range of Y(II). This behavior of vFd against Y(II) was also observed in *crr4* (Figure 5A). Furthermore, vFd showed dependence on the increase in qP (Figure 5B). The increase in qP and the enhancement of PQ oxidation stimulated the appearance of excessive vFd in WT and *crr4*. That is, the activation of photosynthetic linear electron flow oxidized PQ and induced excessive vFd, as observed in the increase in Y(II).

## 3. Discussion

In the present research, we compared the activity of Fd-CEF in *Arabidopsis thaliana,* WT, and *crr4*. There was a nonlinear relationship between Y(II) and vFd (Figure 5A). The gross CO_2_ assimilation rate increased with increasing Ci. On the other hand, Y(II) became saturated. Furthermore, vFd did not saturate and continued to increase (Figure 5A). That is, the increase in vFd deviated from the increase in Y(II) in both WT and *crr4*. This result indicates the presence of excessive vFd unrelated to photosynthetic linear electron flow (Figure 5A). A deviation of vFd from the linear relationship was also found between vFd and qP (Figure 5B). One parameter of Chl fluorescence, qP, reflected the reduction-oxidation state of PQ. The increase in qP showed the oxidation of the reduced PQ, which was induced by the stimulation of the photosynthetic linear electron flow. That is, the appearance of excessive vFd required the reduced PQ to be oxidized.

**Figure 6:**
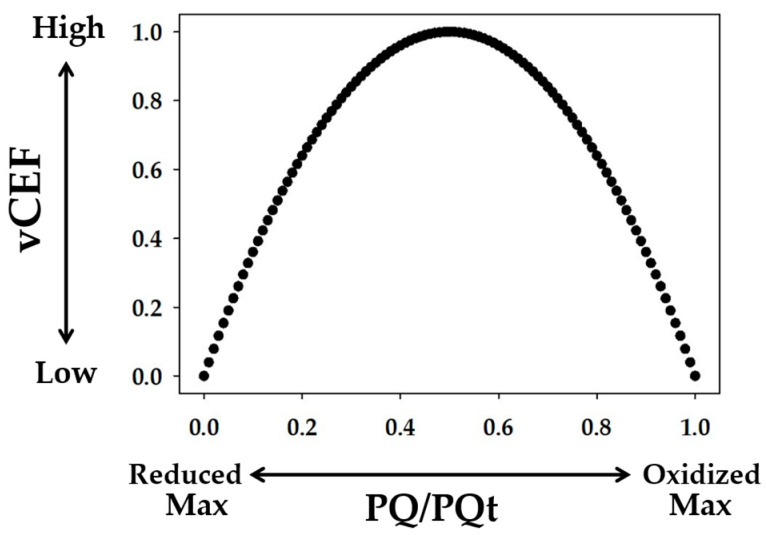
Model for the expression of Fd-CEF activity (vCEF): the dependence of vCEF on the ratio of the oxidized PQ to total PQ pool (PQt), (PQ/PQt). The vCEF is plotted against PQ/PQt according to the model of Allen [1]. In the extremely reduced state of PQ, in which the intensity of actinic light is higher and/or the intercellular partial pressure of CO_2_ is lower, vCEF is greatly suppressed. The stimulation of net CO_2_ assimilation with photosynthetic linear electron flow enhanced the oxidation of the reduced PQ, with vCEF increasing, as shown in the present research.

We propose that the excessive vFd, which is unrelated to the photosynthetic linear electron flow, reflects the electron flux in the ferredoxin-dependent cyclic electron flow around PSI (Fd-CEF), judged from the following model (Figure 6) [1]. In Fd-CEF, the reduced Fd donates electrons to PQ through Fd-quinone oxidoreductase (FQR). That is, the electron donor is the reduced Fd, and the electron acceptor is the oxidized PQ. The Fd-CEF velocity is proportional to the product of the reduced Fd and the oxidized PQ and depends on the activity of FQR [1]. If PQ was completely reduced, the activity of Fd-CEF was zero, even if Fd was reduced (Figure 6). Conversely, if PQ was completely oxidized, the activity of Fd-CEF was also zero because Fd did not possess any electrons for the reduction of PQ [1,5]. In the present research, the reduction-oxidation state of Fd was constant in the range of Y(II) (Figure 2C), due to the oxidation of P700, which reflects the limitation of the electron flow from the reduced PQ to the oxidized P700 in PSI; that is, the reduction of the oxidized P700 is the rate-determining step in the P700 photooxidation reduction cycle [9]. The constant level of reduced Fd induced Fd-CEF activity, depending on the reduced state of PQ (Figure 3B and Figure 5B). As the reduced PQ was oxidized by the stimulation of the linear electron flow of photosynthesis, excessive vFd appeared (Figure 3B and Figure 5B). The reverse was true. These relationships between excessive vFd and qP followed the Fd-CEF model [1]. Hereafter, excessive vFd will be called Fd-CEF activity.

The expression of excessive vFd did not correspond to the enhancement in the ratio of Y(I) to Y(II) caused by the decrease in Y(II) (Supplemental Figure S1A). The increase in the ratio of Y(I) to Y(II) clearly showed a linear relationship with the increase in the oxidation of P700 (Supplemental Figure S1B). The content of the oxidized P700 (P700max’) produced by saturation-pulse light illumination (see Section 4) depended on the rate-determining step of the P700 photooxidation reduction cycle in PSI, and the cycle turned over under saturation-pulse light and actinic light illumination [9]. If the reduction of P700^+^ was the rate-determining step, as observed in the accumulation of the oxidized P700, the yield of P700max’ (Y(I)) became larger than Y(II) and did not reflect the true electron flux in PSI [9].

Fd-CEF activity was not detected in our previous study [13]. The vFd observed during the induction of net CO_2_ assimilation precisely followed the increase in Y(II) in wheat leaves under the higher light intensity. Furthermore, vFd followed Y(II) in the A/Ci analysis under higher light intensity. That is, vFd clearly showed a positive linear relationship with Y(II). In these analyses, Y(II) showed a lower value in the 0.05 to 0.3 range [13]. These lower Y(II) values indicate a higher reduction level of PQ. The present research suggests that higher light intensity and lower Ci induced a higher reduction state of PQ, which suppressed Fd-CEF activity. The expression of Fd-CEF activity requires the oxidation of PQ (Figure 6) [1], which is why we could not detect Fd-CEF activity [13].

It has been proposed that Fd-CEF contributes to excess production of ΔpH across the thylakoid membrane in cooperation with the photosynthetic linear electron flow [2]. The ΔpH induced by Fd-CEF drives the induction of NPQ of Chl fluorescence and satisfies the ATP requirement for achieving a higher rate of net CO_2_ assimilation [1,2]. In the present research, vFd showed a negative relationship with the NPQ of Chl fluorescence (Figure 3A and Figure 5A). However, this finding does not indicate that Fd-CEF cannot induce ΔpH for the induction of NPQ because the excessive vFd was accompanied by the stimulation of the gross CO_2_ assimilation rate as Ci increased (Figure 1 and Figure 5). That is, the increase in ATP consumption occurred simultaneously with the increase in excessive vFd. Therefore, the ΔpH induced by Fd-CEF did not accumulate, or the total ΔpH induced by the enhanced photosynthetic linear electron flow (the light reaction in photosynthesis) driven by net CO_2_ assimilation (the dark reaction in photosynthesis) and the enhanced Fd-CEF (the light reaction in photosynthesis) greatly decreased.

The reaction center Chl in PSI (P700) is oxidized in response to the suppression of net CO_2_ assimilation [17]. This is a universal and robust phenomenon observed in organisms that carry out oxygenic photosynthesis [17,18]. The oxidation of P700 alleviates the accumulation of electrons in the electron acceptors of PSI: Fx, F_A_/F_B_, and Fd [16]. These electron acceptors, including phylloquinone, can donate electrons to O_2_ to produce superoxide anion radicals, the primary product of O_2_ reduction in the Mehler reaction [19,20]. The accumulation of the reduced forms of these electron carriers causes PSI photoinhibition [21,22,23,24,25]; therefore, P700 should be oxidized under the suppressed condition of photosynthesis and/or the suppressed utilization of photon energy for net CO_2_ assimilation [16].

A molecular mechanism has been proposed for the suppression of electron accumulation in the electron carriers at the acceptor side of PSI [17,26]. To suppress the electron accumulation at the acceptor side of PSI, the electron flux to the acceptor side of PSI from PSII should be decreased, and the bottlenecked reaction is the oxidation of reduced PQ by the Cyt *b*_6_/*f* complex. The downregulation of PQ oxidation activity by the Cyt *b*_6_/*f* complex is induced by acidification of the luminal side of the thylakoid membrane [27] and the highly reduced state of PQ (reduction-induced suppression of electron flow, RISE) [17]. RISE occurs under extremely suppressed photosynthetic conditions, in which the induction of acidification at the luminal side of the thylakoid membrane saturates in response to the decrease in photosynthesis activity [28,29]. On the other hand, acidification of the luminal side of the thylakoid membrane, observed as the induction of ΔpH formation across the thylakoid membrane, may be triggered by Fd-CEF [2]. However, as shown in Figure 5, no enhanced activity of Fd-CEF was observed when the efficiency of the net CO_2_ assimilation rate was lower, in which the photosynthetic linear electron flow was suppressed and PQ was highly reduced. That is, Fd-CEF cannot function and cannot contribute to the induction of the ΔpH under such conditions. On the one hand, it was suggested that photorespiration was a prerequisite for the induction of ΔpH formation across the thylakoid membrane [29]. Furthermore, photorespiration can function as soon as actinic light illumination starts [30,31]. The electron flux in the photosynthetic linear electron flow can be accounted for by both net CO_2_ assimilation and photorespiration [17,31]. That is, the photosynthetic linear electron flow, which is observed as Y(II), reflected mainly the net CO_2_ assimilation and photorespiration. Based on this knowledge, it was concluded that unless photorespiration functioned, P700 was not oxidized [32]. Under photorespiratory conditions, Y(II) increased with the enhancement in P700 oxidation as soon as the illumination started with actinic light and without CO_2_ assimilation [32]. Furthermore, photorespiration induced ΔpH formation across the thylakoid membrane, which caused P700 oxidation in rice plants [28]. The rice plants show intrinsic fluctuations in the stomatal opening. This natural phenomenon occurs in all plants [28]. That is, all plants with stomata are exposed to the fluctuating condition of net CO_2_ assimilation, and photorespiratory regulation of the oxidation of the electron carriers at the acceptor side of PSI is necessary at the closed state of the stomata. Then, CO_2_ is decreased at the ribulose 1,5-bisphosphate carboxylase/oxygenase (Rubisco) carboxylation site, and photorespiration is activated to form ΔpH for the suppressed electron flow from PSII to the acceptor side of PSI.

To date, the precise pathway of electron flow in Fd-CEF has not been clarified. In the present research, the possibility of catalyzing Fd-CEF by NDH in *Arabidopsis thaliana* was tested. However, the Fd-CEF activity of the *crr4* mutant, which lacks NDH, was the same as that of WT *Arabidopsis thaliana* (Figure 5). Furthermore, *crr4* showed the same dependence of Fd-CEF activity on Y(II) (Figure 5). That is, NDH is not the mediator for the observed Fd-CEF. The Shikanai group proposed, as a candidate for Fd-CEF, that *pgr5/grl1* proteins mediate Fd-CEF [33,34,35]. However, the mutants deficient in pgr5 and/or pgrl1 showed higher H^+^-conductance, which caused the ΔpH across the thylakoid membrane to decrease and suppress P700 oxidation compared to that of WT [36]. No theory has been demonstrated to explain the higher H^+^-conductance to lower ΔpH across the thylakoid membrane. Recently, it was elucidated that *pgr5*, which the Shikanai group isolated, is a double mutant that showed lower CO_2_ fixation activity than that of WT [37]. That is, the pure single mutant (*pgr5^hope1^*), which is deficient in only pgr5, showed the same electron sink activity and the same CO_2_ fixation rate as WT [16,37]. These results showed that pgr5 is not a factor for Fd-CEF. If pgr5 functioned in Fd-CEF to supply ATP, *pgr5^hope1^* would not maintain the same net CO_2_ assimilation rate as WT. This knowledge should not be ignored when considering the physiological function of pgr5 in photosynthesis. At present, we cannot refer to the physiological function of pgr5 in photosynthesis.

From the knowledge obtained in the present research, one possibility for the molecular mechanism of Fd-CEF in PSI was considered, which does not induce ΔpH formation across the thylakoid membrane [4]. That is, the reduced Fd donates electrons to heme *c* in the Cyt *b*_6_/*f* complex. The reduced heme *c* donates electrons to the high-potential heme *b* through the lower-potential heme *b* in the Cyt *b*_6_/*f* complex. This thermodynamically favored Fd-CEF route drives the fast CEF pathway [4]. If Fd-CEF functioned in the fast CEF pathway, excessive vFd was not related to the induction of NPQ of Chl fluorescence and P700 oxidation in PSI. To further elucidate the physiological function of the excessive vFd and the molecular mechanism driving Fd-CEF, further research is needed.

We developed a method to detect Fd-CEF activity around PSI in intact leaves of *Arabidopsis thaliana*. The oxidation rate of the reduced Fd is a useful indicator of Fd-CEF activity. If the oxidation rate exceeded the photosynthetic linear electron flow rate, Fd-CEF started to function. The Fd-CEF activity required the oxidation of PQ because the reduced Fd could donate electrons to PQ. Therefore, suppression of the net CO_2_ assimilation rate with PQ reduction decreased Fd-CEF activity, in contrast to the expectation that Fd-CEF could induce ΔpH across the thylakoid membrane to regulate the electron flux through PQ to PSI; that is, Fd-CEF could not oxidize P700 to suppress reactive oxygen species (ROS) production in PSI. On the other hand, as the net CO_2_ assimilation increased, Fd-CEF activity increased. The accelerated activity of Fd-CEF would contribute to higher CO_2_ assimilation. Surprisingly, NDH did not affect the activity of Fd-CEF around PSI. Elucidation of the physiological function of NDH requires further research.

## 4. Materials and Methods

### 4.1. Plant Materials and Growth Conditions

*Arabidopsis* plants (*Arabidopsis thaliana* WT and *crr4*) were grown from seeds under standard air-equilibrated conditions with 10 h/14 h day–night cycles at 23 and 20 °C, respectively, and 55–60% relative humidity. The photon flux density was adjusted to 100 μmol photons m^−2^ s^−1^, which was measured with a light meter (LI-189, LI-COR, Lincoln, NE, USA) equipped with a quantum sensor. Seeds were planted in the soil after 3 days of vernalization at 4 °C. Seedlings were kept in 0.2 (dm)^3^ pots containing a 2:1.5 ratio of seeding-culture soil (TAKII Co., Ltd., Kyoto, Japan) to vermiculite and were watered daily. Plants were fertilized with 1000-fold diluted Hyponex fertilizer 8–12–6 (Hyponex Japan, Osaka, Japan) only once in the 3rd week after seeding. The plants at 10 h after the dark duration start of the light/dark cycle of growth conditions were used for all the measurements, which were conducted using rosette leaves of 5- to 6-week-old plants.

### 4.2. Determination of Chlorophyll and Nitrogen

Leaves after measuring photosynthetic parameters were sampled for content analysis and stored at −80 °C until use. Upon sampling, detached leaves were weighed and electronic images were acquired with a scanner for leaf area measurement by ImageJ (NIH). The Chl and nitrogen (N) contents in the leaves of WT and *crr4* were determined [38] and are shown in Supplemental Table S1. A raw leaf blade was homogenized in 50 mM sodium-phosphate buffer (pH 7.2) containing 120 mM 2-mercaptoethanol, 1 mM iodoacetic acid, and 5% (*v*/*v*) glycerol at a leaf:buffer ratio of 1:9 (g/mL) in a chilled mortar and pestle. The total Chl and leaf N contents were measured from a part of this homogenate. The absorbance at 663.6 and 646.6 nm was measured to calculate the Chl content [39]. The Chl content in the leaves is represented on a leaf-area basis [38]. The total leaf N content was determined using Nessler’s reagent in a digestion solution after potassium sodium tartrate was added [38]. The homogenate was decomposed by 60% (*v*/*v*) sulfuric acid and 30% (*v*/*v*) H_2_O_2_ with heat. The decomposing leaf solution was mixed with distilled water, 10% (*w*/*v*) potassium sodium tartrate solution, and 2.5 N NaOH, and Nessler’s reagent was immediately added to the mixture. The N content was determined by measuring the change in absorbance at 420 nm.

### 4.3. Simultaneous Measurements of Chlorophyll Fluorescence, P700, and Fd-Signals with Gas Exchange

Chl fluorescence, P700, Fd, and CO_2_ exchange were simultaneously measured using Dual/KLAS-NIR (Heinz Walz GmbH, Effeltrich, Germany), and an infrared gas analyzer (IRGA) LI-7000 (Li-COR, Lincoln, NE, USA) measuring system equipped with a 3010-DUAL gas exchange chamber at several ambient partial pressures of CO_2_ at 21 kPa O_2_ (Heinz Walz GmbH) was used [16]. The gases were saturated with water vapor at 16 ± 0.1 °C. The leaf temperature was controlled at 25 ± 0.5 °C (relative humidity: 55–60%). The actinic photon flux density at the upper position on the leaf in the chamber was adjusted to the indicated intensity. The net CO_2_ assimilation rate (A) and the dark respiration rate (Rd) were measured.

The Chl fluorescence parameters were calculated as follows [40]: F_o_, minimum fluorescence from a dark-adapted leaf; F_o_′, minimum fluorescence from a light-adapted leaf; F_m_, maximum fluorescence from a dark-adapted leaf; F_m_′, maximum fluorescence from a light-adapted leaf; Fs, fluorescence emission from a light-adapted leaf; the apparent quantum yield of PSII, Y(II) = (F_m_′ − Fs)/F_m_′ [41]; non-photochemical quenching, non-photochemical quenching (NPQ) = (F_m_ − F_m_’)/F_m_’ [42]; and PQ oxidized state (qP) = (F_m_’ − Fs)/(F_m_’ − F_o_’) [43]. To obtain F_m_ and F_m_′, a saturating pulse light (630 nm, 8000 µmol photons m^−2^ s^−1^, 300 ms) was applied. Red actinic light (630 nm, 400 µmol photons m^−2^ s^−1^) was supplied using a chip-on-board LED array.

The signals for oxidized P700 (P700^+^), oxidized plastocyanin (PC^+^), and reduced ferredoxin (Fd^−^) were calculated based on the deconvolution of four pulse-modulated dual-wavelength difference signals in the near-infrared region (780–820, 820–870, 840–965, and 870–965 nm) [44]. Both P700 and PC were completely reduced, and Fd was fully oxidized in the dark. To determine the total photo-oxidizable P700 (P700max) and PC (PCmax), a saturation flash was applied after 10 s of illumination with far-red light (740 nm). The following formulas were used: The apparent quantum yield of PSI, Y(I) = (P700max’ − P700^+^)/P700max; the quantum yield of oxidized P700 (P700^+^), Y(ND) = P700^+^/P700max; and the apparent quantum yield of nonphotochemical energy dissipation of photoexcited P700 (P700*), Y(NA) = (P700max − P700max′)/P700max. In the present research, we showed Y(ND) as P700^+^ (Figure 2A). The summation of these quantum yields is 1 (Y(I) + Y(ND) + Y(NA) = 1). Total photo-reducible Fd (Fdmax) was determined by illumination with red actinic light (450 µmol photons m^−2^ s^−1^) after plant leaves were adapted to the dark for 5 min [44]. The redox states of both P700 and PC under actinic light illumination were evaluated as the ratios of P700^+^ and PC^+^ to total P700 and total PC, respectively. The redox state of Fd was also determined similarly. The values of P700max, PCmax, and Fdmax in the leaves of WT and crr4 are shown as relative values in Supplemental Table S1.

For the analysis of dark-interval relaxation kinetics (DIRK analysis, [45]), red actinic light (400 µmol photons m^−2^ s^−1^) was temporarily turned off for 400 ms at steady-state photosynthesis [13]. The oxidation rate of Fd^−^ was estimated by a Dual/KLAS-NIR spectrophotometer and expressed as the relative values by estimating the initial decay of Fd^−^.

### 4.4. Statistical Analysis

Statistical analyses of the corresponding data in Supplemental Table S1 (CI, confidential interval) were performed using the commercial software JMP8 (ver. 14.2.0, SAS Institute Inc., Cary, NC, USA).

## Data Availability

Data are contained within the article and Supplementary Material.

## Supplementary Materials

The following supporting information can be downloaded at https://www.mdpi.com/article/10.3390/ijms241512145/s1.
