# Supplementary material for: Evaluating the Oxidation Rate of Reduced Ferredoxin in Arabidopsis thaliana Independent of Photosynthetic Linear Electron Flow: Plausible Activity of Ferredoxin-Dependent Cyclic Electron Flow around Photosystem I
Source: Int J Mol Sci. 2023 Jul 29;24(15):12145. doi: 10.3390/ijms241512145 (PMC10419245; doi:10.3390/ijms241512145)
Supplement: Supplementary file 1 [file ijms-24-12145-s001.zip › ijms-2472207-supplementary.pdf]

Supplemental Materials:

Supplemental Table S1

	Chl (mmol m^{-2})			N (mmol m^{-2})			Fv/Fm			P700max (Relative value)			PCmax (Relative value)			Fdmax (Relative value)		
Field	mean	SD	CI	mean	SD	CI	mean	SD	CI	mean	SD	CI	mean	SD	CI	mean	SD	CI
WT	0.366	0.036	difference	47.4	5.7	difference	0.799	0.002	difference	3.74	0.32	difference	1.96	0.24	difference	0.54	0.08	difference
crr4	0.318	0.018	(-0.1038, 0.0087)	50.7	3.2	(-5.77, 12.30)	0.799	0.005	(-0.00850, 0.00973)	3.27	0.21	(-0.955, 0.030)	1.71	0.06	(-0.636, 0.146)	0.46	0.01	(-0.2005, 0.0405)

CI, Confidential Interval of the difference between WT and crr4 (95%); SD, Standard Deviation.

Supplemental Figure S1

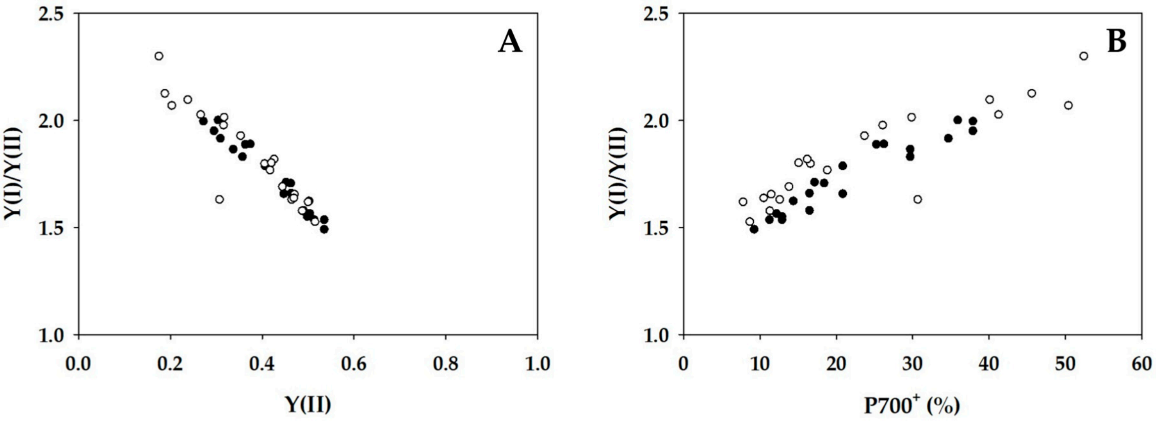

Relationships between Y(I)/Y(II), Y(II), and P700$^+$. The data for each parameter were measured in the experiments depicted in Figure 1, simultaneously with the net CO_2 assimilation rates and Y(II). (**A**) Y(II) and (**B**) P700$^+$ were plotted against Y(I)/Y(II). The data were obtained from four independent experiments using leaves attached to four WT and *crr4* plants ($N = 4$). Black symbols, WT; White symbols, *crr4*.
